# Deep reconstruction model for dynamic PET images

**DOI:** 10.1371/journal.pone.0184667

**Published:** 2017-09-21

**Authors:** Jianan Cui, Xin Liu, Yile Wang, Huafeng Liu

**Affiliations:** 1 State Key Laboratory of Modern Optical Instrumentation, Department of Optical Engineering, Zhejiang University, Hangzhou, China; 2 Shenzhen Institutes of Advanced Technology, Chinese Academy of Sciences, Shenzheng, China; Chongqing University, CHINA

## Abstract

Accurate and robust tomographic reconstruction from dynamic positron emission tomography (PET) acquired data is a difficult problem. Conventional methods, such as the maximum likelihood expectation maximization (MLEM) algorithm for reconstructing the activity distribution-based on individual frames, may lead to inaccurate results due to the checkerboard effect and limitation of photon counts. In this paper, we propose a stacked sparse auto-encoder based reconstruction framework for dynamic PET imaging. The dynamic reconstruction problem is formulated in a deep learning representation, where the encoding layers extract the prototype features, such as edges, so that, in the decoding layers, the reconstructed results are obtained through a combination of those features. The qualitative and quantitative results of the procedure, including the data based on a Monte Carlo simulation and real patient data demonstrates the effectiveness of our method.

## Introduction

Positron emission tomography (PET) is a nuclear medicine, functional imaging method that evaluates the body condition based on metabolism in living tissue. Annihilation occurs due to the decay of a radio tracer such as ^18^F-Fluoro-2-Deoxy-glucose (FDG) after emission positron encounters the electrons, which generates a pair of 511keV photons in the opposite direction. Once the projection data are obtained by a photon detector, the static radioactivity distribution can be reconstructed [1]. In contrast to static PET imaging, dynamic PET imaging detects data in a series of frames. In clinical applications, the dynamic information has the potential to improve early detection, the characterization of cancer and assessment of therapeutic response after obtaining the spatial and temporal radioactivity distribution [2]. To guarantee that the rapid change in tracer activity can be tracked immediately, the time interval between two frames in earlier parts of a scan will not be long enough, leading to reduced photon accumulation and lower spatial resolution. How to reconstruct the high-quality dynamic PET images and guarantee the temporal resolution at the same time has become a challenging problem [3].

One popular method is to reconstruct the radioactivity map for each frame independently based on several conventional statistical models such as maximum a posteriori (MAP) [4], maximum likelihood estimate (MLE) [5] and penalized weighted least square (PWLS) [6]. Because of the tradeoff between spatial resolution and temporal resolution, these frame-to-frame strategies may lead to noisy results due to the low signal-to-noise ratio of the data. To solve this problem, an attractive approach is to append the smoothness regularization terms into the objective function. Compared with other approaches such as the filtering of wavelets [7], total variation (TV) regularization can be suitable for edge-preserving imaging problems in low signal to noise ratio (SNR) or few-view data sets; however, in the presence of noise, TV tends to over smooth and deblur images with a natural gradient [8].

In this study, we aimed to reduce the noise while preserving the key features (such as the boundary of a tumor) at the same time. Different from regularizations in the above methods, we built a deep reconstruction framework for PET images based on the stacked sparse auto-encoder (SAE) and the maximum likelihood expectation maximization (MLEM), which is called MLEM+SAE in short. The SAE model comprised several auto-encoder templates, where each template exploited self-similarity in PET images. To incorporate the information in adjacent frames, temporal features were also learned by the single-layer sparse auto-encoder. The essence of our MLEM+SAE model was to incorporate the PET images feature automatically and boost the reconstruction accuracy.

## Methods

The study was approved by the Research Ethics Committee of Zhejiang University. The patients provided written informed consent.

### Dynamic PET imaging model

After injecting the radio tracer into the body, the detected emission data represented the series of photon pairs. The relationship between the detected photon pairs and radioactive distribution could be written as:

$$\begin{array}{c} y_{i_{q}}∼Poisson({\bar{y}}_{i_{q}})s.t.{\bar{y}}_{i_{q}}=\sum_{p}G_{pq}x_{i_{p}} \end{array}$$ (1)

where $x_{i_{p}}$ is the number of photons emitted from source voxel *p* for the *i*th frame, and *G*_*pq*_ is the probability that a photon emitted from voxel *p* is detected at detector *q*. The emission data $y_{i_{q}}$ is the number of photons detected at projection detector *q* for the *i*th frame. The overline for $y_{i_{q}}$ indicates the expectation number of photon detections. For dynamic PET, the whole collected emission data *Y* was modeled by:

$$\begin{array}{c} Y=GX+E \end{array}$$ (2)

where *Y*^*T*^ = [*y*_1_, *y*_2_⋯*y*_*N*_] is the collection of whole emission data, *X*^*T*^ = [*x*_1_, *x*_2_⋯*x*_*N*_] is the collection of original radioactive distribution images, and *E*^*T*^ = [*e*_1_, *e*_2_⋯*e*_*N*_] is the collection of noise in total *N* frames.

### Dynamic PET reconstruction framework based on SAE

**Fig 1:**
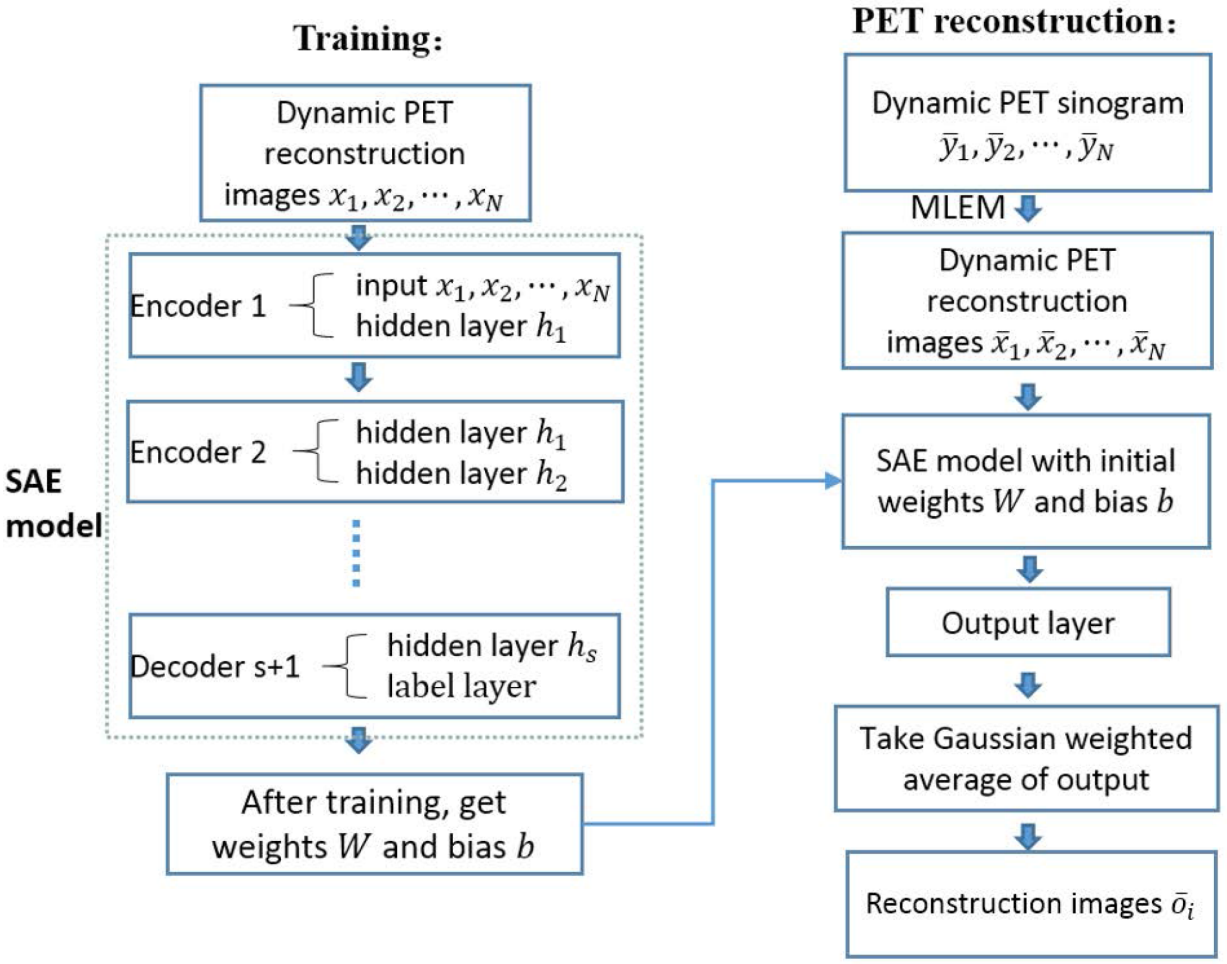
Overflow of the dynamic PET image reconstruction framework.

Our whole framework is summarized in Fig 1. Considering that images in different frame followed the same biological metabolism process, we considered the adjacent reconstruction images as prior knowledge to aid in the reconstruction of other data. The whole framework included two parts, training and reconstruction. During the training step, the series of reconstruction images *x*_1_, *x*_2_⋯*x*_*N*_ were considered the input of a SAE model. The SAE model was combined by several encoders and a decoder that using the ground truth of PET images in a selected *i*th frame as a label. After training, we got weights parameter *W* and bias parameter *b*. In the reconstruction step, we firstly reconstructed the dynamic PET images ${\bar{x}}_{1},{\bar{x}}_{2},\ldots ,{\bar{x}}_{N}$ from sinograms ${\bar{y}}_{1},{\bar{y}}_{2},\ldots ,{\bar{y}}_{N}$ by MLEM, and then we acquired the reconstruction images ${\bar{x}}_{1},{\bar{x}}_{2},\ldots ,{\bar{x}}_{N}$ as input importing into the SAE model with the corresponding parameter *W* and *b* that have been trained in the training step. The estimate value of PET images in the *i*th frame could be calculated from the Guassian weighted average of the output. The details are elaborated in the following section.

#### Training data

Training data in the form of a carefully chosen set of images could be used to represent samples of the prior, such as texture for the desired solution. Clearly, such images must be reliable and application specific.

#### Auto-encoder template and SAE model

Generally speaking, directly performing medical image analysis is challenging because the dimensions of the medical images are usually large and the structure of the biological tissue is complex. Traditional unsupervised learning methods such as principal component analysis (PCA) [9] and sparse dictionary learning [10] are widely used for data dimension reduction; However, both methods represent a single-layer feature. To address the above problem, we constructed the auto-encoder structure to infer the low-level feature in a lower dimension; indeed, the auto-encoder can be stacked layer by layer and learn the intrinsic hierarchical feature representations [11].

**Fig 2:**
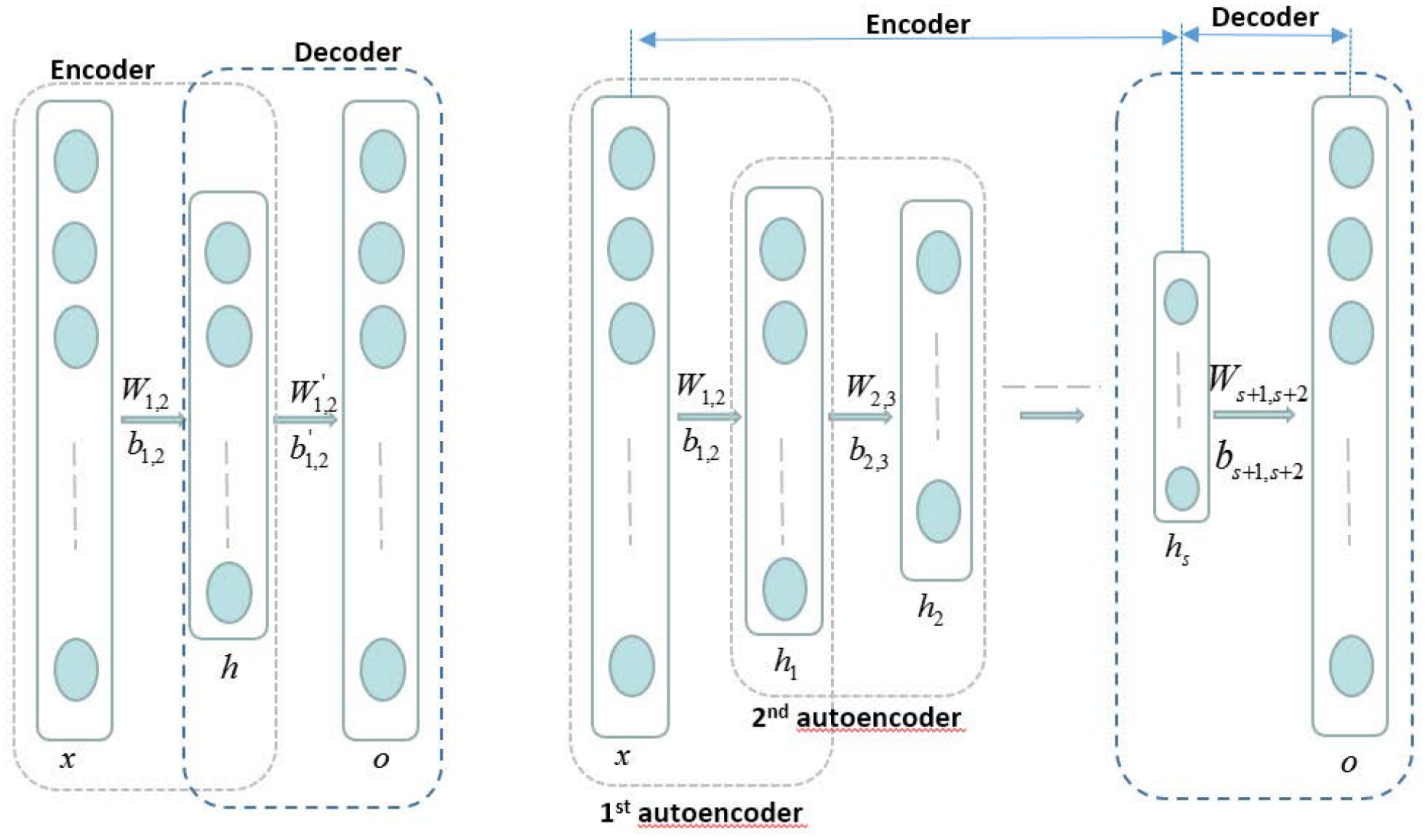
Left: Auto-encoder template. Right: SAE model. An autoencoder is a three layer network including an encoder and a decoder. The SAE model is combined by several encoders and a decoder. The hidden layer of an encoder is the input of its next encoder.

Fig 2 (left) shows the auto-encoder template. An autoencoder is a three layer network including an encoder and a decoder. We firstly reshaped the gray-level pixels of the two dimension reconstruction image as the column vector, considered input *x*, and then obtained the output *o* by the encoding and decoding processes:

$$\begin{array}{c} h=\sigma (W_{1,2}x+b_{1,2}) \end{array}$$ (3)

$$\begin{array}{c} o=\sigma (W_{1,2}^{{}^{\prime }}h+b_{1,2}^{{}^{\prime }}) \end{array}$$ (4)

The objective function is:

$$\underset{W_{1,2},b_{1,2},W_{1,2}^{{}^{\prime }},b_{1,2}^{{}^{\prime }}}{\text{min}}{\left\|o-x\right\|}^{2}+\alpha_{1}{\left\|W\right\|}_{F}+\alpha_{2}KL\left(\rho_{W}∥\rho_{0}\right)$$ (5)

In the above equation, *x*, *h* and *o* represent the input layer, hidden layer and output layer, respectively. *σ* is the sigmoid function, and *W*_1,2_, *b*_1,2_, $W_{1,2}^{{}^{\prime }}$, $b_{1,2}^{{}^{\prime }}$ are the encoding weight, encoding bias, decoding weight and decoding bias, respectively. *α*_1_ and *α*_2_ are weighting parameters. ‖*W*‖_*F*_ is the sum of the Frobenius norms of all of the weight matrices to regularize the value of the matrix elements. *KL*(*ρ*_*W*_ ∥ *ρ*_0_) is the penalty item of the Kullback-Leibler divergence between the weight matrix sparsity and the sparsity set in advance:

$$\begin{array}{c} KL(\rho_{W}∥\rho_{0})=\sum_{ij}\rho_{W}\log\frac{\rho_{W}}{\rho_{0}}+(1-\rho_{W})\log\frac{1-\rho_{W}}{1-\rho_{0}} \end{array}$$ (6)

The first term in Eq (5) is the penalty for loss function; using this term, the auto-encoder template actually learns a function *F*(*W*, *b*) that is an approximation to the identity function. In other words, after learning the weights and bias, the calculated results are similar to the original input:

$$\begin{array}{c} F(W_{1,2},b_{1,2},W_{1,2}^{{}^{\prime }},b_{1,2}^{{}^{\prime }},x)\approx x \end{array}$$ (7)

**Fig 3:**
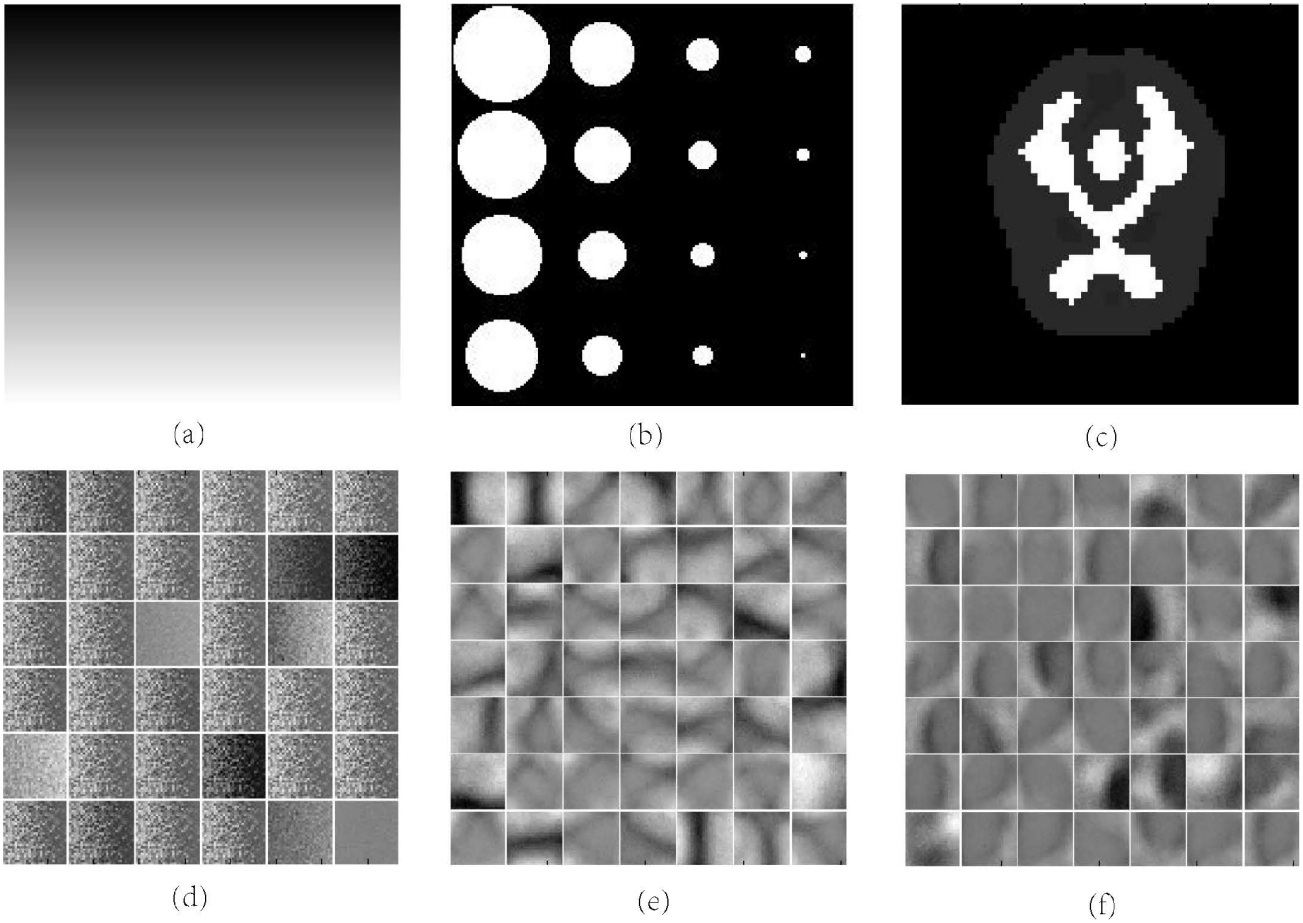
Visualization of filters learned with SAE. (a) (b) Different physical phantoms. (d) (e) Different features learned from above phantoms. (c) Brain phantoms. (f) Features learned from brain phantoms.

The encoding step is to learn the feature from the original input where the basic elements are gray-level pixels, and this particular structure makes the input compressed as represented by the learned features after setting the number of hidden nodes less than the input layer. Through visualizing the linear weights in the input-to-first layer weight matrix, which is also called “filters”, the features extracted by the first layer can be represented directly. For the smooth area, the representation are sparse; on the other hand, the representation could be dense if the certain patch has rich information or complex structure. Fig 3 shows two physical phantom (a, b) and its visualization of corresponding filters (d, e) where the features are the patches of gradually changed pixels in line and circular curves. The second term in (5) is a regularization of the values for each parameter to avoid over fitting, and the last term actually decide how much of the main feature is retained; the sparsity setting *ρ*_0_ is larger when more features are retained.

After initializing all of the parameters, we updated the weights and bias using the back propagation algorithm [12]. The iteration progress gradually achieved convergence after multiple training samples. The information about the original input image is encoded in the hidden layer vector, which has learned the low-level feature of the original image into a lower dimension space. Fig 3(f) shows the features of the brain phantom learned with SAE.

Similarly, we designed several auto-encoder templates and stacked them together to learn more detailed features. The input layer of the next template is the hidden layer of the previous one. The whole framework is shown in the right of Fig 2.

Given the series of reconstruction images for dynamic PET imaging *x*_1_, *x*_2_⋯*x*_*N*_, we designed an SAE framework. First, we reshaped all of the adjacent reconstruction images *x*_1_, *x*_2_⋯*x*_*N*_ to a column vector and considered them the input of the first auto-encoder template. After solving the parameters *W*_1,2_ and *b*_1,2_, we calculated the hidden layer *h*_1_ by Eq (3) and considered it the input of the second auto-encoder template. After several iterations, we obtained the whole SAE. Next, we added the output layer after the last hidden layer, where the nodes of output layer are the same as the dimension of the *i*th frame image. Using the back propagation algorithm and ground truth of the middle frame *i* as a label, we computed the final parameters *W*_*s*+1,*s*+2_ and *b*_*s*+1,*s*+2_.

### Implementation

#### Model initialization

The first preparation was to initialize the parameters to be solved in the above auto-encoder templates. Random initialization does not always produce the optimal solution due to following reasons. First, greater weights easily lead to the local optimal solution; on the other hand, the gradient may vanish during the back propagation with smaller weights, a phenomenon called gradient diffusion. To avoid those situations, we adopted the restricted Boltzmann machine (RBM) to initialize the parameters and solve it using the contrastive divergence algorithm [13].

**Fig 4:**
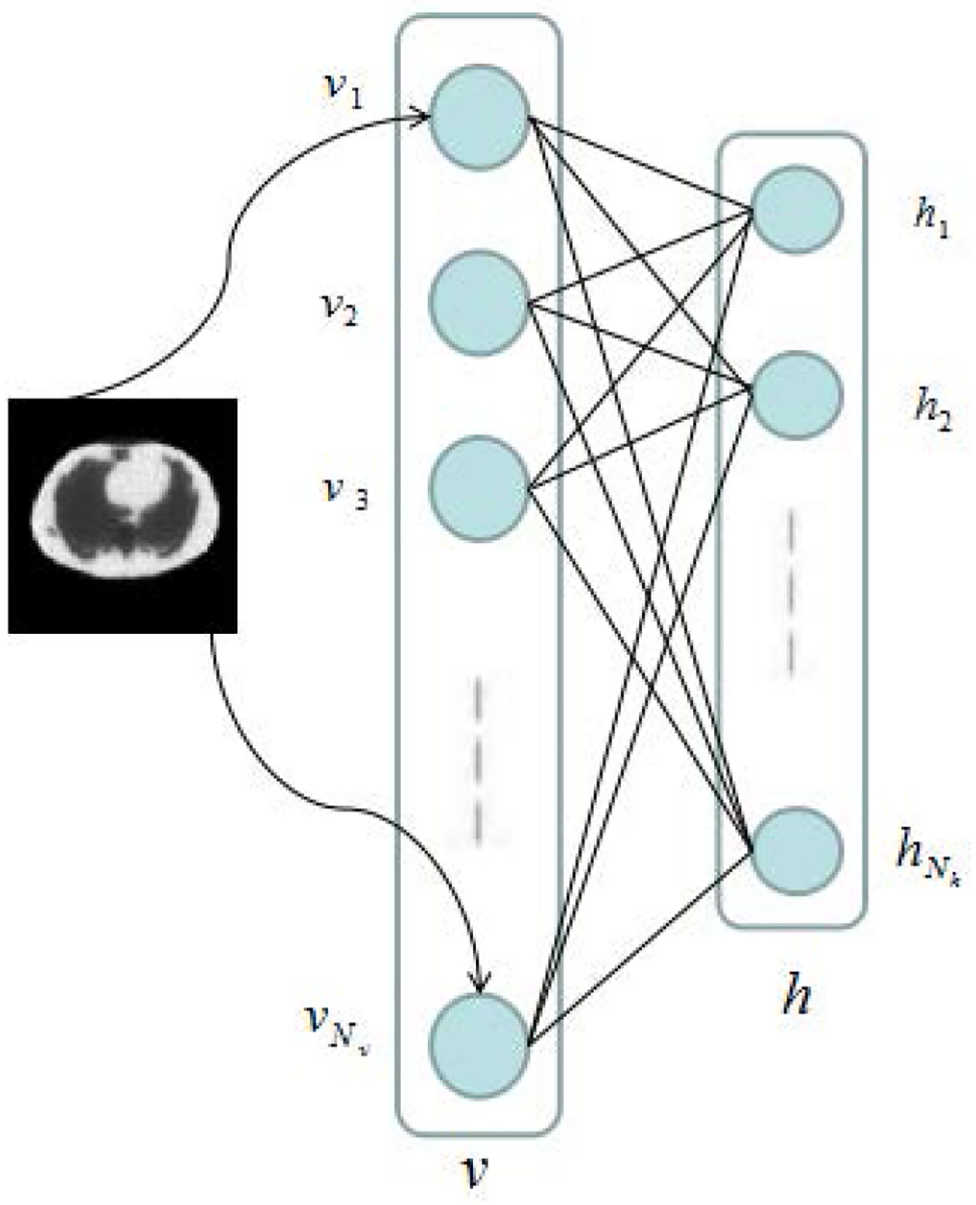
Restricted Boltzmann machine.

As shown in Fig 4, the restricted Boltzmann machine comprises a visible layer{*v*_*i*_}, *i* = 1, 2, …, *N*_*v*_ and a hidden layer {*h*_*j*_}, *j* = 1, 2, …, *N*_*h*_. *W* is the *N*_*x*_ by *N*_*h*_ matrix connecting the visible layer to the hidden layer, and the joint probability density of RBM is:

$$\begin{array}{c} P(v,h)=\frac{1}{Z}\exp(-E(v,h)) \end{array}$$ (8)

where *Z* is the constant of normalization, *E*(*v*, *h*) is the energy function defined as:

$$\begin{array}{c} E(v,h)=\sum_{i}b_{i}v_{i}-\sum_{j}c_{j}h_{j}-\sum_{ij}w_{ij}v_{i}h_{j} \end{array}$$ (9)

where *b* and *c* are the biases of the visible layer and hidden layer, respectively. By maximizing the likelihood function, we can train the parameters *θ* = (*W*, *b*, *c*).

First, because there is no connection between nodes from the same layer, we can rewrite the joint probability density function as follow:

$$\begin{array}{c} P_{\theta }(v,h)=\frac{1}{Z(\theta )}\prod_{ij}e^{W_{ij}v_{i}h_{j}}\prod_{i}e^{b_{i}v_{i}}\prod_{j}e^{c_{j}h_{j}} \end{array}$$ (10)

Thus, we obtain the condition probabilities:

$$\begin{array}{c} \begin{array}{c} P\left(h_{j}=1|v\right)=\frac{1}{1+\text{exp}\left(-\sum_{i}W_{ij}v_{i}-c_{j}\right)}\\ P\left(v_{i}=1|h\right)=\frac{1}{1+\text{exp}\left(-\sum_{j}W_{ij}h_{j}-b_{i}\right)} \end{array} \end{array}$$ (11)

Given a training sample {*v*_1_, *v*_2_⋯*v*_*N*_} to satisfy the independent distribution, we need to maximize the following likelihood function:

$$\begin{array}{c} L\left(\theta \right)=\frac{1}{N}\sum_{n=1}^{N}\text{log}P_{\theta }\left(v_{n}\right)-\frac{\lambda }{N}{\left\|W\right\|}_{F}^{2} \end{array}$$ (12)

where λ is the relative coefficient between the data items and regularization items. After calculating the gradient of likelihood function to the parameters, we can solve the problem using the gradient descent algorithm.

#### Parameter training

In the actual experiment for dynamic PET reconstruction, we did not consider the whole image in the vector of input but only a small patch for the following reasons. First, the dimension of the medical image is very large: assuming the size of one image is *l* by *k*, the total nodes in the input layer is *l* × *k* × *N*, and too many input nodes makes the training process become slow and difficult to obtain an accurate solution. Second, because of the limitation of the number of training images, dividing the images into patches will increase the sample quantity, avoiding under fitting. Algorithm 1 shows the detailed steps.

**Algorithm 1. SAE Parameters Training**

1. **Initialize** the number of frames *N*, reconstruction frame *i*, number of hidden layers *s*, number of nodes in each of the hidden layers *h*_1_, *h*_2_⋯*h*_*s*_, and size of patch *l* × *k*.

2.  **For** each PET phantom:

3.  simulate the corresponding dynamic PET emission data *y*_1_, *y*_2_⋯*y*_*N*_.

4.  obtain the reconstruction images *x*_1_, *x*_2_⋯*x*_*N*_ from the emission data.

5.   **For** each patch in *x*:

6.   consider the pixels of patches as the input and initialize the parameters *W*_1,2_, *b*_1,2_ using the RBM.

7.   update the parameters *W*_1,2_, *b*_1,2_ using the back propagation algorithm.

8.   calculate the hidden layer and consider it the next input of the auto-encoder.

9.   perform steps 6-8 until *W*_1,2_, *b*_1,2_, ⋯, *W*_*s*,*s*+1_, and *b*_*s*,*s*+1_ have been updated completely.

10.   initialize and update *W*_*s*+1,*s*+2_, *b*_*s*+1,*s*+2_ using the ground truth of the *i*th frame.

11.   **END**

12.  **END**

13. **Return:***W, b*

#### PET image reconstruction

Given the new dynamic PET emission data, we firstly reconstructed the original images, selected a patch position and applied all of the patches to the input layer, and calculated the output vector through the trained parameters *W* and *b*. Finally, we considered Gaussian average of the output vector the estimate value of the center of the patches for the middle frame *i*. By changing the position and sweep through all of the patches, we obtained the whole estimate image, which was the *i*th frame PET image reconstruction result. The details are illustrated in Algorithm 2.

**Algorithm 2. SAE PET image reconstruction**

1. **Initialize** emission data.

2. Reconstruct the original radioactive distribution images by MLEM.

3.  **For** each pixel position (*r*, *s*):

4.  select the *l* × *k* patch in each reconstruction image.

5.  reshape all of the patches into the column vector and apply it to the input layer.

6.  calculate the output layer through the trained parameters *W* and *b*.

7.  consider the Gaussian weighted average of the output as the estimate value.

8.  **END**

9. **Return:** the *i*th frame PET image reconstruction image

## Experiments

To confirm the accuracy and effectiveness of our method, we conducted several experiments using Monte Carlo simulated data and real patient data, respectively. We compared our method (shorthand for MLEM+SAE) with the traditional algorithm (MLEM) and total variation (TV) regularization based method. All of the codes were implemented and run in MATLAB R2014a (MathWorks Corporation, USA) and personal computer with i5 Intel Core CPU and 8GB memory.

In this paper, we used the following three quantitative indexes for comparison:

$$\begin{array}{c} \begin{array}{c} SNR=20\times \log(\frac{255}{\sqrt{\frac{1}{n}\sum_{i=1}^{n}{\left(u_{i}-{\hat{u}}_{i}\right)}^{2}}})\\ Bias=\frac{1}{n}\sum_{i=1}^{n}(\frac{u_{i}-{\hat{u}}_{i}}{{\hat{u}}_{i}})\\ Variance=\frac{1}{n}\sum_{i=1}^{n}{\left(\frac{u_{i}-{\bar{u}}_{n}}{{\hat{u}}_{i}}\right)}^{2} \end{array} \end{array}$$ (13)

where *u*_*i*_ is our reconstruction value in the *i*th pixel, and ${\hat{u}}_{i}$ is value of ground truth in the *i*th pixel. ${\bar{u}}_{n}$ is the mean value and *n* is the number of pixels in interest region. The SNR reflects the accuracy of the reconstructed image: a higher SNR indicates better results. The bias and variance reflect the difference between the result and ground truth: a lower bias or variance indicates better results.

### Parameter setting

Before the experiments, we initialized our model with the number of frames *N* = 3, number of hidden layers *s* = 2, number of nodes in the hidden layers *h*_1_ = 200, *h*_2_ = 100, and size of patches *l* × *k* = 7 × 7. The aim of our model is to find the relation on temporal to help reconstruction. For dynamic PET imaging, the previous frame and the next frame are the most closely associated with the to be reconstructed frame. So we set *N* = 3 and in the input layer of our model is the *i* − 1th, *i*th and *i* + 1th frame. Usually, the number of hidden layers in lots of stacked autoencoder applications is set as two to four [14] [15] [16]. As our model is a low-level vision task, which emphasizes more pixel level features, too deep network is not needed. Therefore the number of hidden layers is set as *s* = 2. The number of nodes in the input layer is decided by the defined patch size: small patches carry less information, and large patches make the whole framework more difficult to train. By contrast, the total number of nodes in the hidden layers decides the learning ability of the framework: fewer nodes lead to under fitting, and too many nodes make training difficult and promote over fitting. A different setting will influence the quality of the final results, and all of the parameters are decided by multiple tests. For the framework of SAE, we considered two parameters, focusing on their complexity and capability, which are the total number of nodes in the input layer and hidden layer, respectively.

**Fig 5:**
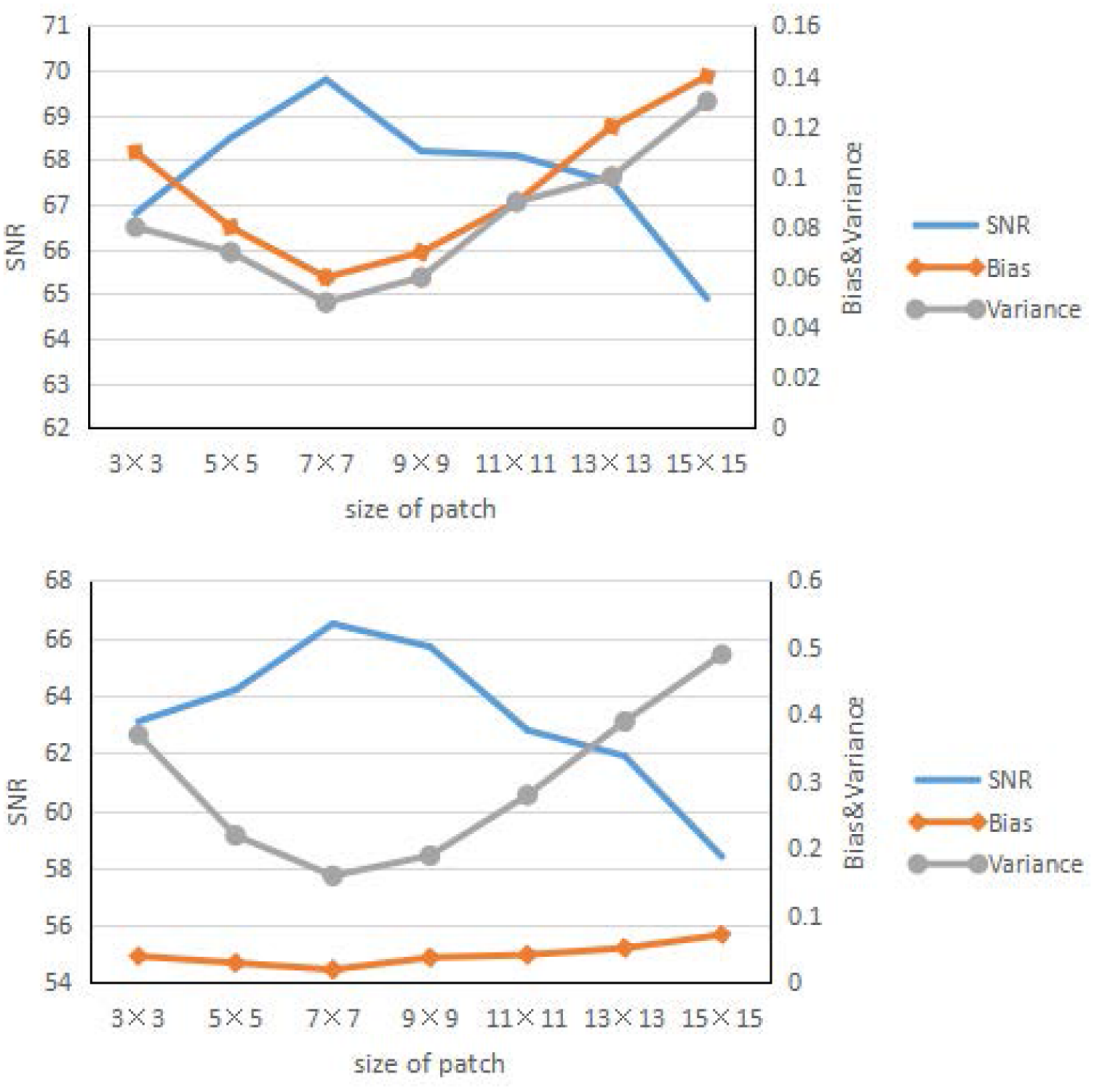
Reconstruction results. Reconstruction results of the brain phantom (left) and Zubal phantom (right) for different size of patches.

**Fig 6:**
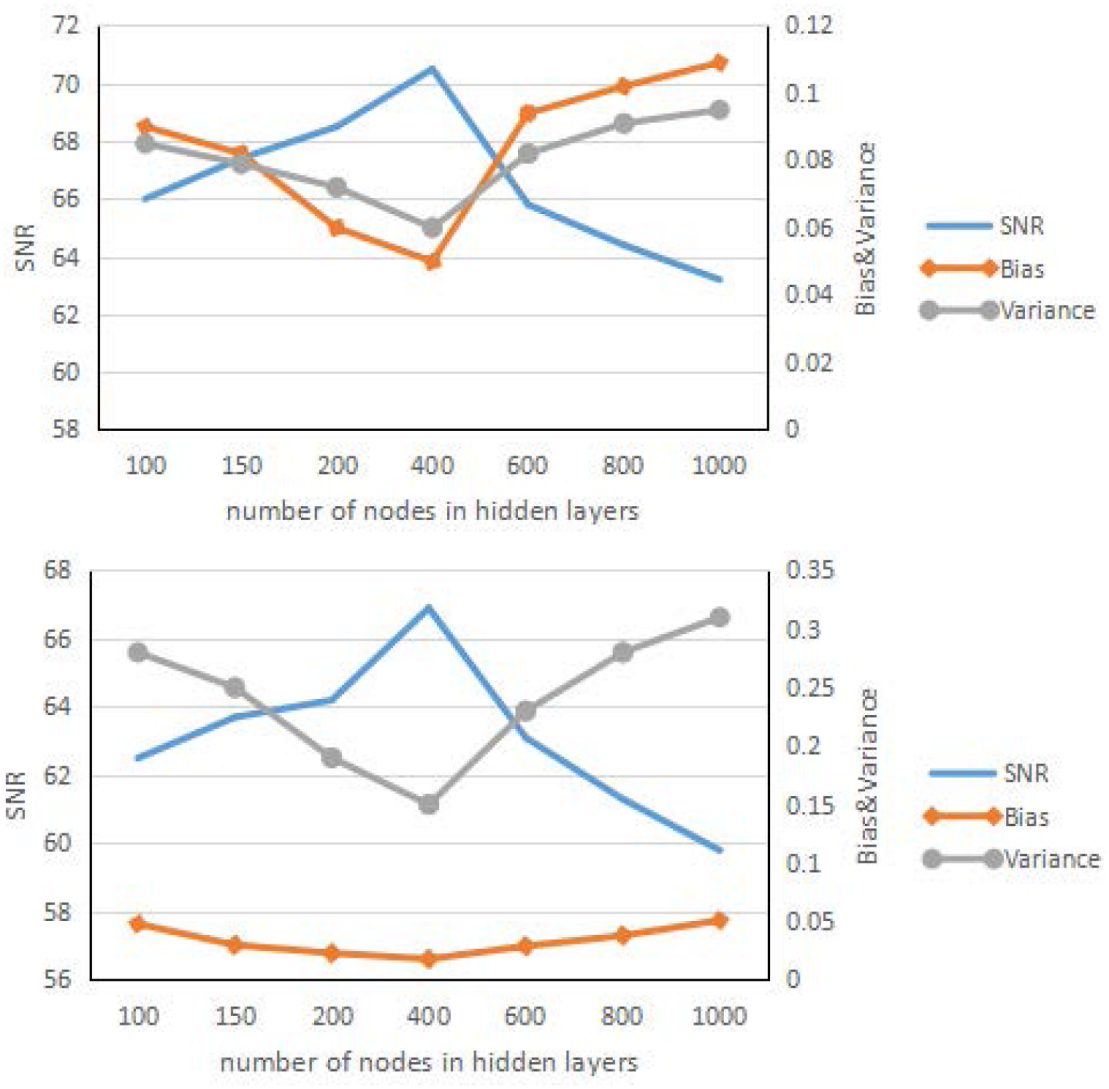
Reconstruction results. Reconstruction results of the brain phantom (left) and Zubal phantom (right) for different numbers of nodes in the hidden layers.

Figs 5 and 6 show the result of the influence of the patch size and total number of nodes in the hidden layers. These two figures have some similarities, at the beginning; as the patch size and number of nodes increase, the learning ability of the framework becomes stronger, and the corresponding SNR results improve. After a certain time, the results for the larger patch worsen, the framework shows over fitting, and the corresponding SNR results worsen quickly. Overall, the best patch size was approximately 7 × 7, and the number of nodes in the hidden layer is approximately 400.

### Accuracy

#### Monte Carlo simulation

**Fig 7:**
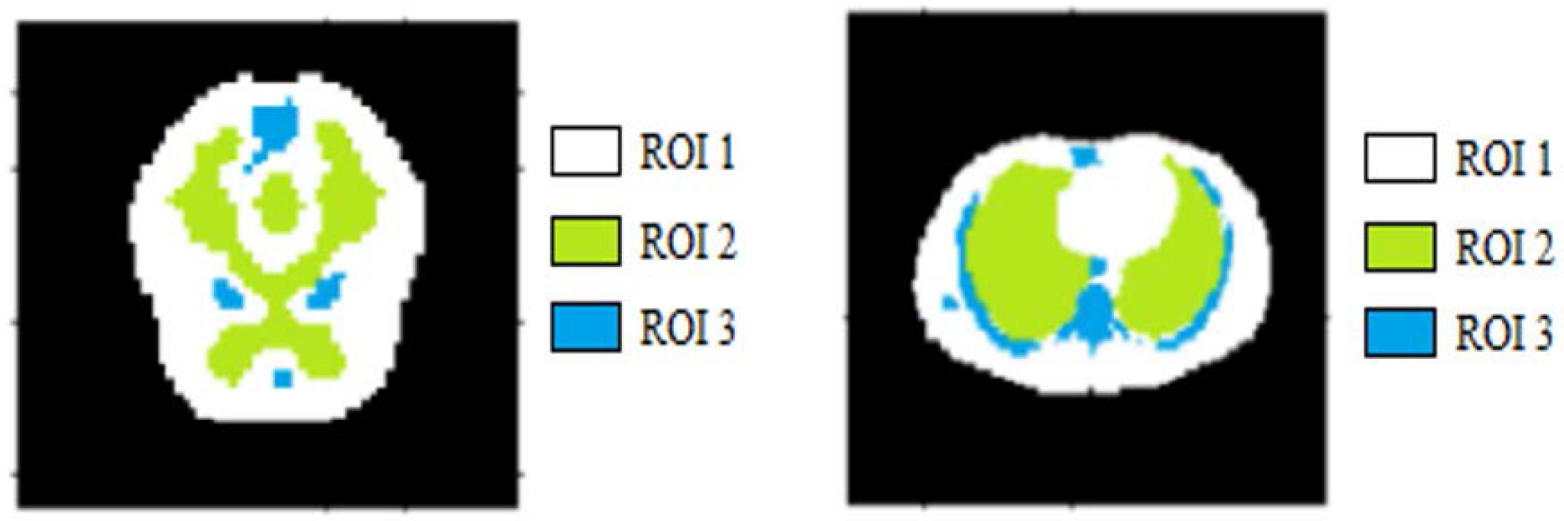
Monte Carlo simulation results. Monte Carlo simulation brain phantom data (left) and Zubal phantom data (right).

We conducted our experiment for the brain phantom [17] and Zubal phantom [18] using the toolbox GATE [19] to perform Monte Carlo simulation.The benefits of Monte Carlo simulation are that we could simulate physical and physiological processes to compare our method with other reconstruction algorithms and the ground truth. In this paper, the dynamic PET scanner simulated was Hamamatsu SHR74000, and the corresponding radio tracer was ^18^*F* − *FDG*. The sinogram of the brain phantom had 64 × 64 projections, and the number of frames was 18. The sinogram of Zubal phantom had 128 × 128 projections, and the number of frames was 16. We also obtained data with different counting rates-i.e., the count number of coincidence events set as 5 × 10^4^, 1 × 10^5^, 5 × 10^5^ and 1 × 10^6^. During reconstruction, we also considered random correction, attenuation correction, scatter correction and normalization correction. The ground truth and different ROIs for the brain phantom and Zubal phantom are shown in Fig 7. The network was trained on 700 sets of Monte Carlo simulated data and tested on 100 sets of Monte Carlo simulated data. Each set had more than 20000 patches.

**Fig 8:**
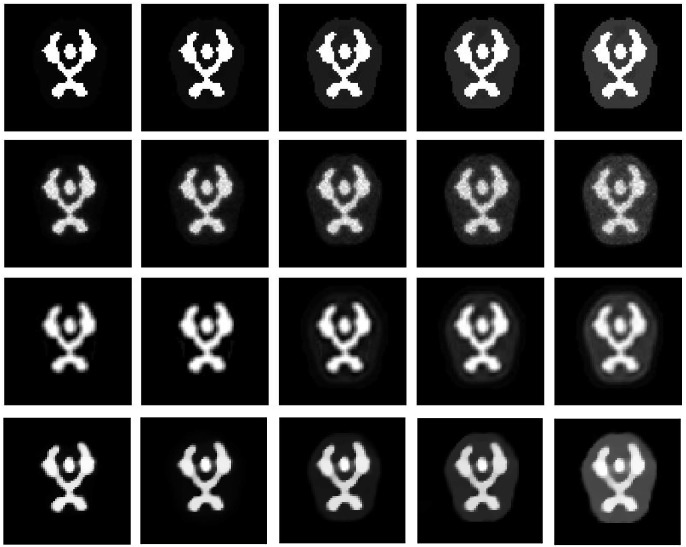
Brain phantom reconstruction results. From top to bottom: ground truth, reconstruction result by MLEM, MLEM+SAE and TV. From left to right: the 1st, 3rd, 5th, 7th, and 9th frames.

**Fig 9:**
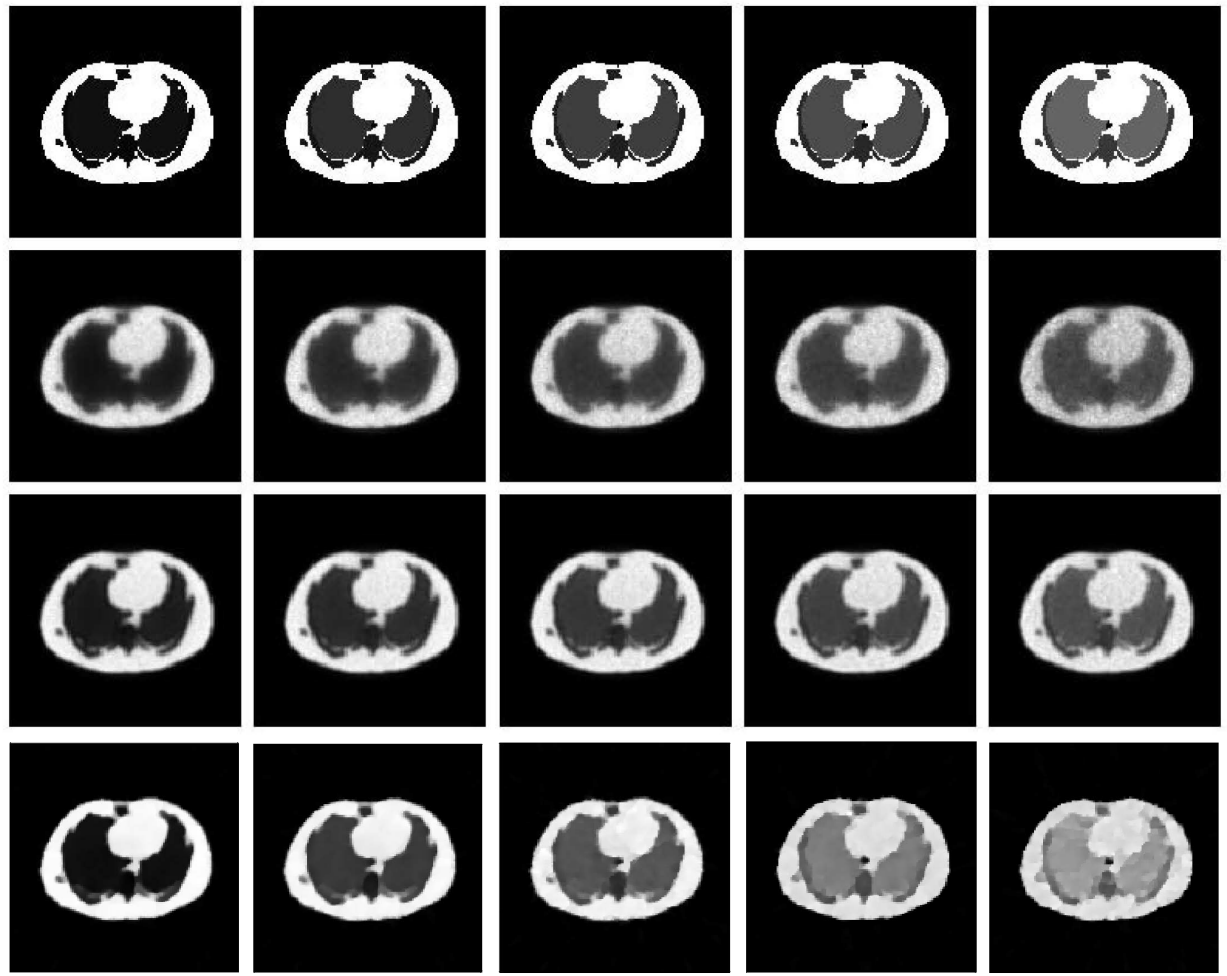
Zubal phantom reconstruction results. From top to bottom: ground truth, reconstruction result by MLEM, MLEM+SAE and TV. From left to right: the 1st, 3rd, 5th, 7th, and 9th frames.

**Fig 10:**
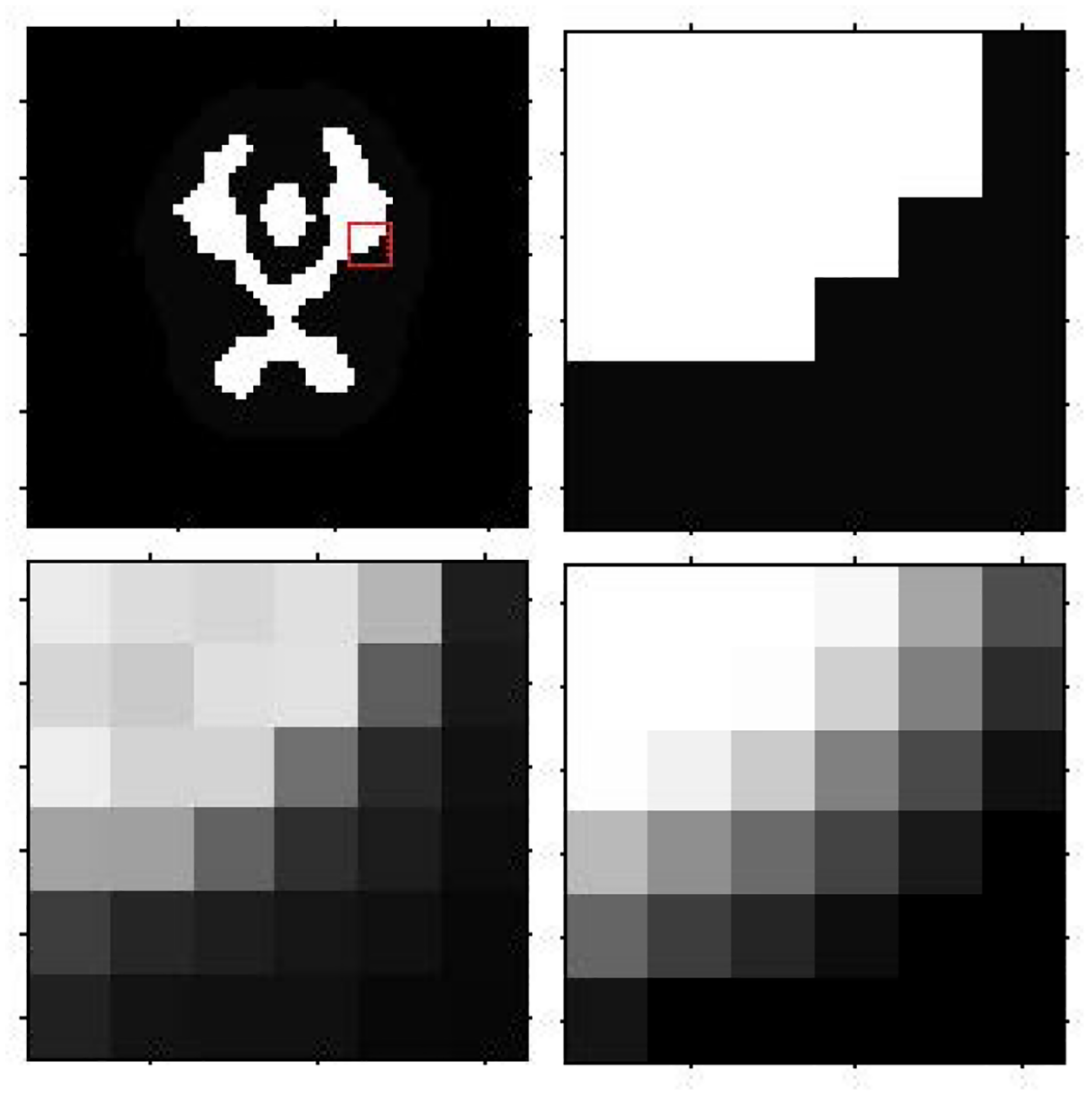
Brain phantom reconstruction results for the local patch. First row: ground truth for the 3rd frame, ground truth for the local patch. Second row: reconstruction result for local patch by MLEM, reconstruction result for local patch by MLEM+SAE.

**Fig 11:**
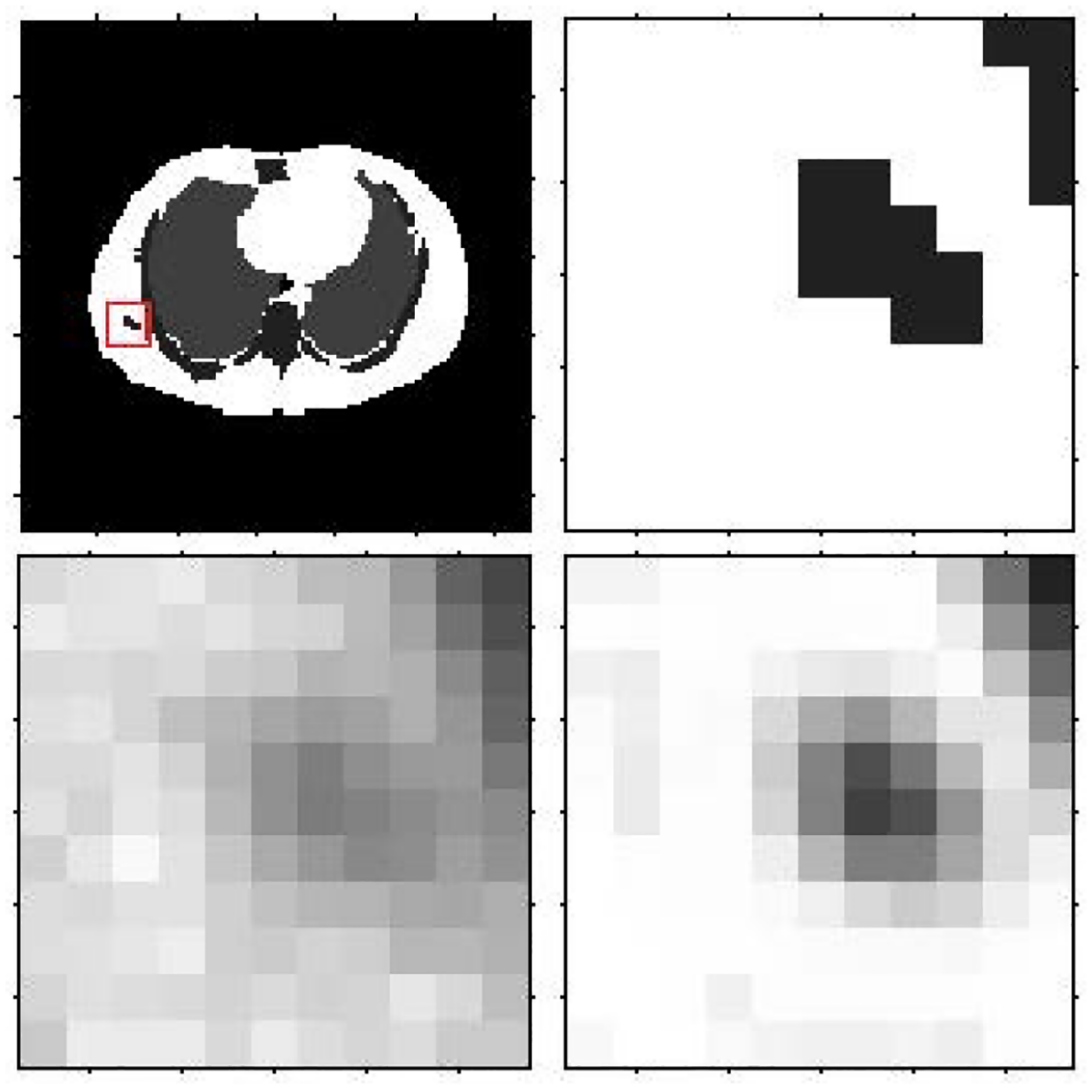
Zubal phantom reconstruction results for the local patch. First row: ground truth for the 3rd frame, ground truth for the local patch. Second row: reconstruction result for local patch by MLEM, reconstruction result for the local patch by MLEM+SAE.

Figs 8 and 9 show the reconstruction results by MLEM, MLEM+SAE and TV for the different brain phantom and Zubal phantom frames. Figs 10 and 11 display selected areas of the reconstruction results by MLEM and MLEM+SAE. From the images, the reconstruction results of our method demonstrated sharper edges and higher pixel values in the radiation area. The MLEM algorithm leads to much noise due to the chessboard effect during iterations; however, by fusing multiple image data, our method provides a clearer and smoother result. The reconstruction results by TV show a good performance when the photon counting rates are high in the 1st, 3rd and 5th frames, but when in the 7th and 9th frames, there are much noise which leads to over smooth and fuzzy boundaries. However, the reconstruction process of our method does not smooth the boundary because the SAE learns the features of the edges and corners in images, resulting in more image details.

**Fig 12:**
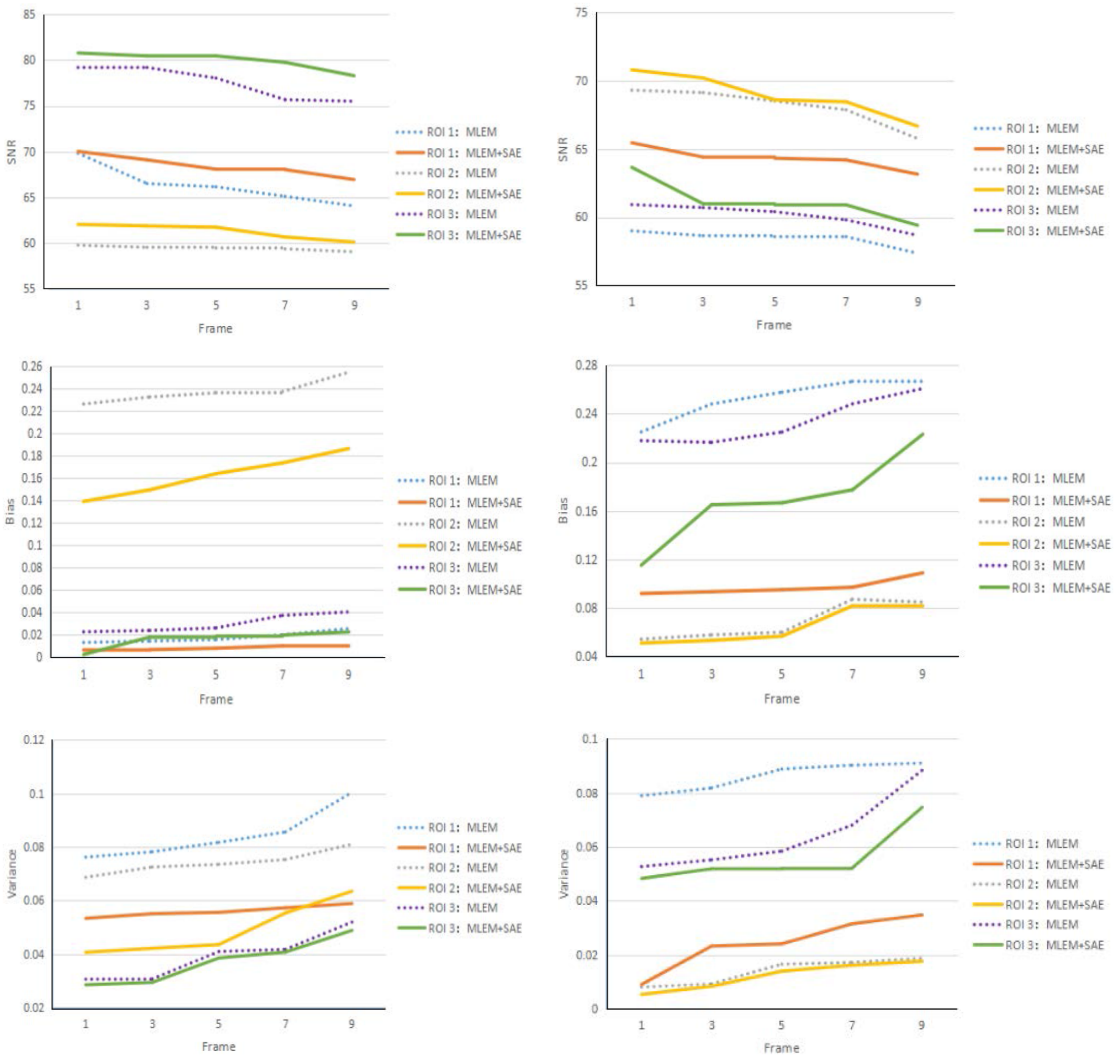
Brain phantom and Zubal phantom reconstruction result. Brain phantom (left) and Zubal phantom (right) reconstruction result comparison for different regions of interest. From top to bottom: SNR, bias and variance comparison curves.

Different regions in our simulated data have different physiological and physical properties that may lead to different reconstruction results. For further analysis, we also performed comparisons among different regions of interest. Fig 12 shows the results of SNR, bias and variance comparison among different regions of interest. From Fig 12, we can conclude that, regardless of the region of interest, our method can improve the SNR and suppress bias and variance in all of the frames.

#### Real data

**Fig 13:**
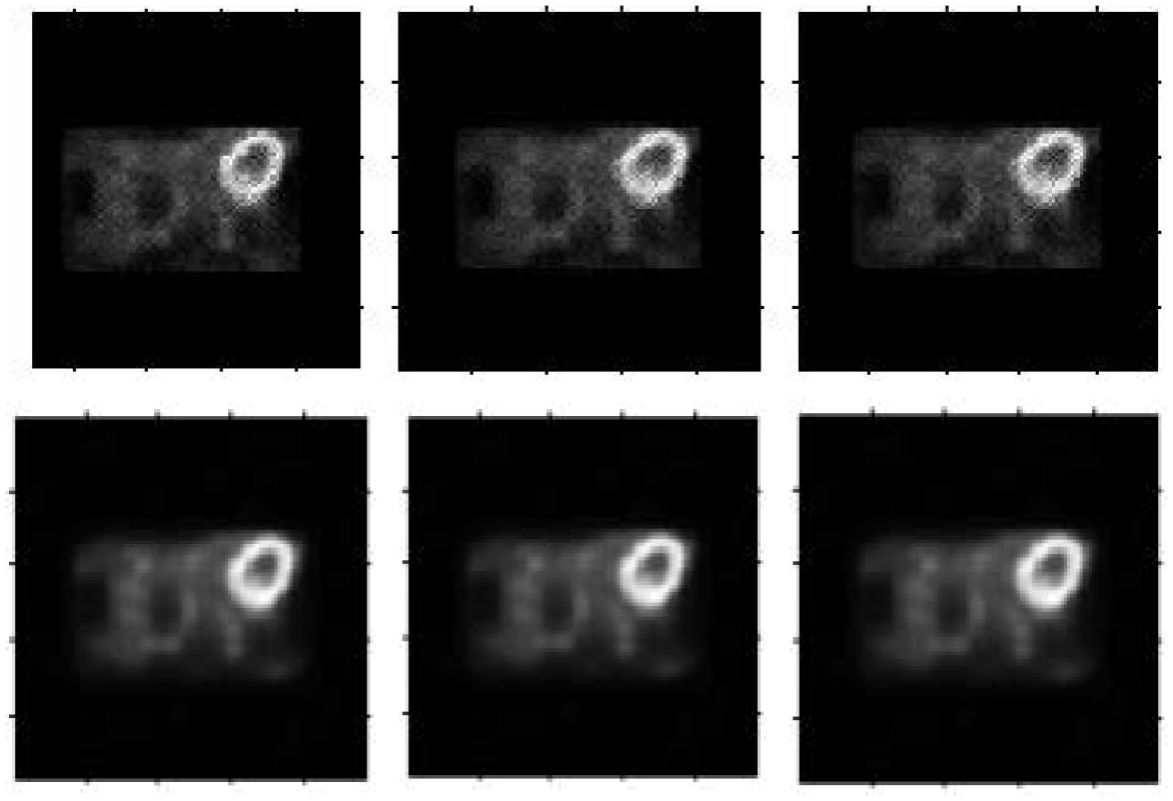
Real heart data reconstructed results. From left to right: Reconstruction result by the MLEM algorithm for the 2nd, 3rd and 4th frames, our result for the 2nd, 3rd and 4th frames.

The patient heart data were obtained from the local hospital using a Hamamatsu SHR-22000 whole-body PET scanner, which has 32 crystal rings and a trans axial resolution of the central FOV of 3.7 mm/p. The size of the collected sinogram is 96 × 96 × 18. The reconstructed results are shown in Fig 13.

From the real patient data reconstruction results, much noise appears in the results using the MLEM algorithm again. By fusing the series of frames, our method produces a cleaner and smoother result. Moreover, the high-concentration radio region remains bright and distinct, and the boundary stays sharper. In summary, our results are more suitable for clinical application obviously.

### Robustness

**Fig 14:**
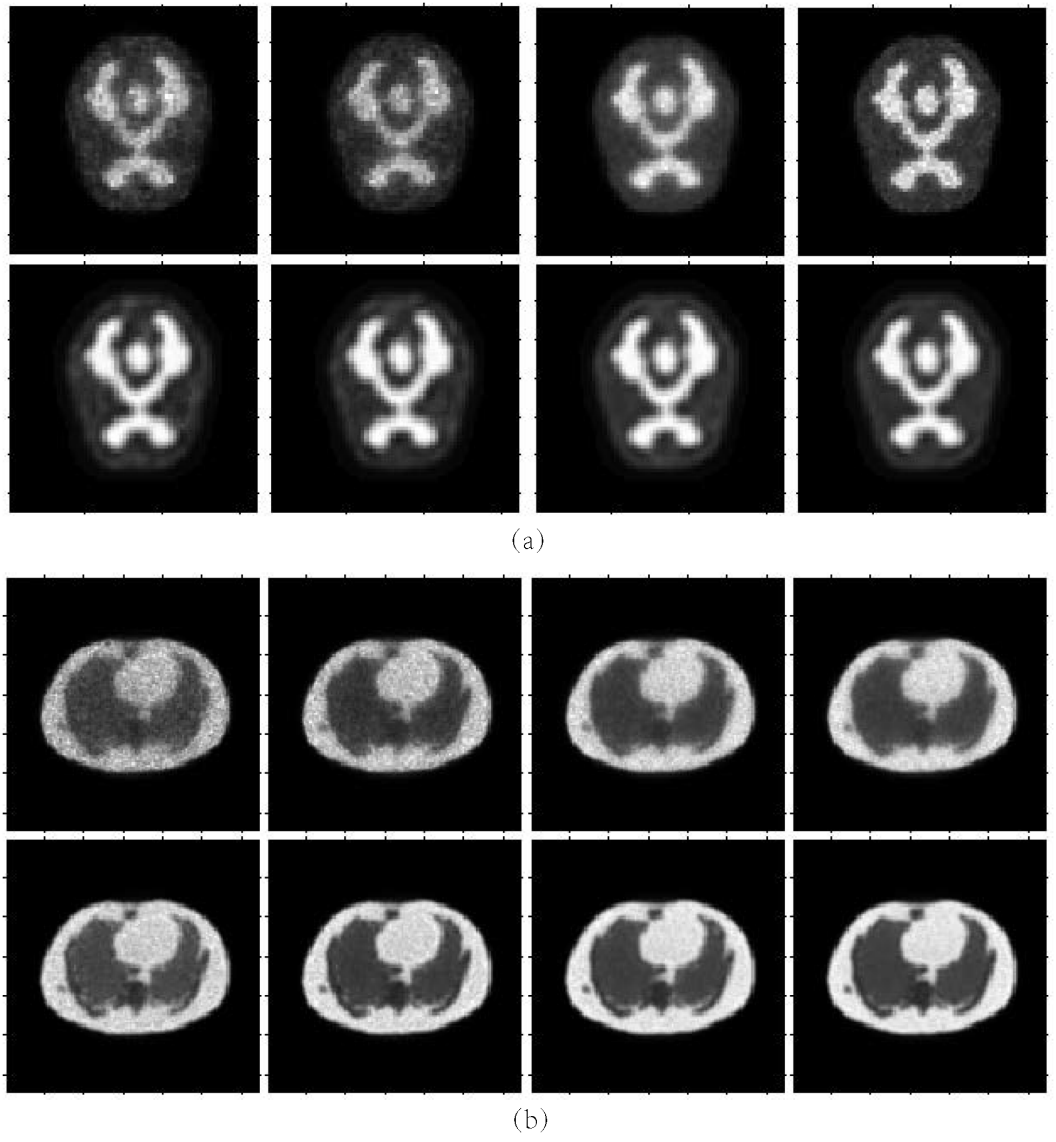
Reconstruction results under different counting rate settings. From left to right: the counting rates are 5 × 10^4^, 1 × 10^5^, 5 × 10^5^ and 1 × 10^6^. Top row: reconstruction results by MLEM. Second row: reconstruction results by MLEM+SAE. (a) Brain phantom. (b) Zubal phantom.

To test the robustness of our results, we set different counting rates-i.e., the number of coincidence events during Monte Carlo simulation. Fig 14 shows the reconstruction result under different counting settings. The detail index comparisons are illustrated in Tables 1 and 2. From these figures and tables, we can conclude that the MLEM algorithm causes much more noise when the counting rate is low: the quality of the reconstruction image improves with the increasing counting rate. However, by learning how to fuse the adjacent reconstruction images, our method can also produce a high-accuracy reconstruction result with much less noise and a clearer boundary.

**Table 1:** Brain phantom reconstruction results comparison with different counting rates.

Counting Rate	ROI	SNR		Bias		Variance	
		MLEM	MLEM+SAE	MLEM	MLEM+SAE	MLEM	MLEM+SAE
5 × 10^4^	Total	66.39	69.30	0.0516	0.0409	0.0146	0.0076
	ROI 1	66.49	68.98	0.0860	0.0715	0.0148	0.0082
	ROI 2	56.58	61.70	0.3588	0.1638	0.1428	0.0439
	ROI 3	76.01	76.37	0.0317	0.0295	0.0016	0.0014
1 × 10^5^	Total	65.76	69.20	0.0548	0.0410	0.0172	0.0078
	ROI 1	66.28	69.18	0.0872	0.0721	0.0153	0.0077
	ROI 2	55.83	61.66	0.3952	0.1639	0.1698	0.0443
	ROI 3	76.65	77.37	0.0290	0.0272	0.0014	0.0012
5 × 10^5^	Total	67.69	69.60	0.0434	0.0378	0.0100	0.0071
	ROI 1	66.54	67.69	0.0780	0.0737	0.0144	0.0111
	ROI 2	59.04	62.21	0.2559	0.1533	0.0809	0.0391
	ROI 3	73.19	77.88	0.0484	0.0251	0.0031	0.0011
1 × 10^6^	Total	69.17	70.42	0.0387	0.0365	0.0079	0.0073
	ROI 1	66.97	69.81	0.0782	0.0573	0.0132	0.0068
	ROI 2	59.75	61.73	0.2325	0.1640	0.0687	0.0436
	ROI 3	79.57	80.19	0.0218	0.0162	0.0027	0.0017

**Table 2:** Zubal phantom reconstruction results comparison with different counting rates.

Counting Rate	ROI	SNR		Bias		Variance	
		MLEM	MLEM+SAE	MLEM	MLEM+SAE	MLEM	MLEM+SAE
5 × 10^4^	Total	63.09	68.06	0.0901	0.0428	0.0319	0.0101
	ROI 1	55.78	64.62	0.3973	0.0995	0.1716	0.0224
	ROI 2	66.25	67.03	0.1138	0.0950	0.0154	0.0129
	ROI 3	58.57	64.62	0.2391	0.1280	0.0904	0.0224
1 × 10^5^	Total	64.21	68.16	0.0766	0.0395	0.0247	0.0099
	ROI 1	56.76	63.83	0.3486	0.1050	0.1370	0.0267
	ROI 2	68.28	69.97	0.0712	0.0643	0.0097	0.0065
	ROI 3	59.50	62.25	0.2108	0.1798	0.0729	0.0387
5 × 10^5^	Total	65.34	68.41	0.0661	0.0369	0.0190	0.0094
	ROI 1	58.14	64.41	0.2887	0.0962	0.0997	0.0235
	ROI 2	67.91	71.12	0.0607	0.0581	0.0105	0.0050
	ROI 3	59.45	61.17	0.2109	0.2083	0.0737	0.0497
1 × 10^6^	Total	63.09	68.06	0.0901	0.0428	0.0319	0.0101
	ROI 1	55.78	64.62	0.3973	0.0995	0.1716	0.0224
	ROI 2	66.25	67.03	0.1138	0.0950	0.0154	0.0129
	ROI 3	58.57	64.62	0.2391	0.1280	0.0904	0.0224

## Discussion and conclusion

We have developed an SAE model for dynamic PET reconstruction. Compared with the existing method, our algorithm outperforms in three main aspects: i)by using the multiple frames as the input, much noise is smoothed and suppressed in the non-radioactivity area and continuous radioactivity area; ii) due to the multi-layer model for feature learning, our algorithm can recover many more details in the boundary or complex area; iii) by processing the image patch by patch, our algorithm can work on dynamic PET emission data regardless of the original size. To demonstrate the effectiveness of our method, we have tested our algorithm for different numerical indexes based on Monte Carlo simulation data and real patient data.

**Fig 15:**
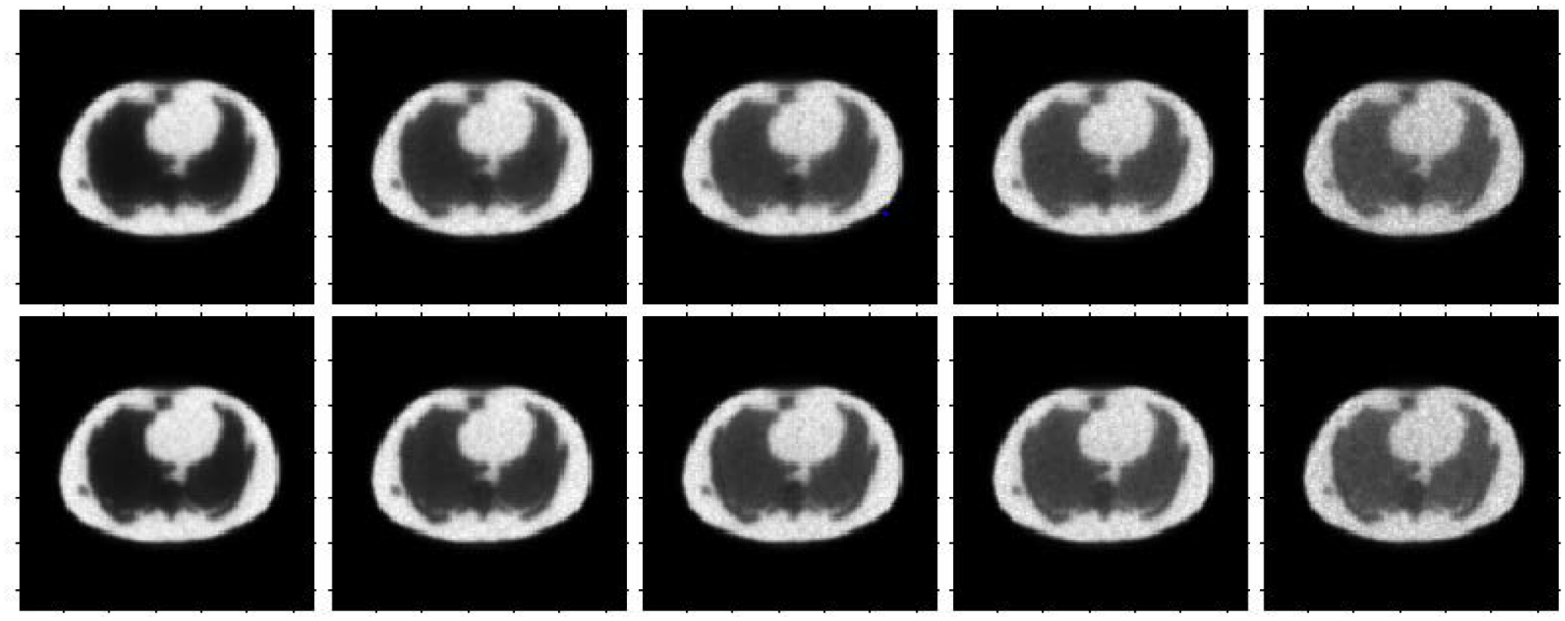
Reconstruction results for the Zubal phantom data. Reconstruction results for the Zubal phantom data using the MLEM algorithm (top row) and MLEM+SAE (second row) with a brain phantom for training. From left to right: the 1st, 3rd, 5th, 7th, and 9th frames.

One of the major concerns is the tissue specificity or patient specificity. Because our parameters are trained in advance, our model may not learn the features when they come from new different test tissue PET images, which will influence the quality of the reconstruction images. As shown in Fig 15, the top row represents the reconstruction results by the MLEM algorithm for the Zubal phantom, and the bottom row represents the reconstruction results by MLEM+SAE; however, we only used the patches of the brain phantom in the training step. From the figure, our method still produces better results than the traditional MLEM algorithm; however, due to the limitation of the training data, the results are not as good as our results in Fig 9, regardless of the noise reduction or edge preservation. Table 3 shows the detailed index comparisons where our method could effectively obtain a higher signal to noise and suppress the bias and variance. With the development of computer ability and appearance of increasing medical data, we believe a stronger computation model can be pro-posed.

**Table 3:** Zubal phantom reconstruction results comparison.

	Method	Frame				
		1st	3rd	5th	7th	9th
**SNR**	MLEM	65.9133	65.8159	65.7752	65.4952	64.7382
	MLEM+SAE(b)	66.3655	66.2882	66.1905	65.8829	65.5591
	MLEM+SAE	68.7277	68.5393	68.4180	68.1678	67.5108
**Bias**	MLEM	0.0587	0.0609	0.0618	0.0628	0.0743
	MLEM+SAE(b)	0.0549	0.0566	0.0589	0.0597	0.0677
	MLEM+SAE	0.0353	0.0356	0.0361	0.0371	0.0393
**Variance**	MLEM	0.0167	0.0170	0.0172	0.0183	0.0218
	MLEM+SAE(b)	0.0150	0.0153	0.0156	0.0168	0.0181
	MLEM+SAE	0.0087	0.0091	0.0094	0.0099	0.0115

MLEM+SAE(b) is the method that only uses the brain phantom for the training step.

In contrast to the traditional reconstruction algorithm that works on the emission data directly, our method needs an initial training step, and then, reconstruction is performed for multiple frames in the reconstruction step. Thus, our method requires much more computational time. Usually, the MLEM algorithm needs 0.8-1.5 seconds for convergence, but our method needs approximately 40-50 seconds, including training and reconstruction. When the data and image dimension increase, we can use more advanced optimization methods, such as batch acceleration gradient reduction, or more complicated structures, such as a convolution neural network. Regarding the hardware, the graphic processing unit can promote high computational efficiency.
